# Role of Long Non-Coding RNAs in Food Wanting of *Apis Mellifera*

**DOI:** 10.3390/insects16121214

**Published:** 2025-11-28

**Authors:** Zun Wu, Hangyu Zhang, Shuting Pu, Linfeng Li, Zhaoyang Zeng, Yang Lü, Zhuo Chen, Xueling Xu, Songkun Su

**Affiliations:** 1 College of Animal Sciences, Fujian Agriculture and Forestry University, Fuzhou 350002, China; zunwu126@163.com; 2 College of Bee Science, Fujian Agriculture and Forestry University, Fuzhou 350002, China; 3 Heilongjiang Academy of Agricultural Sciences, Mudanjiang 157000, China

**Keywords:** dopamine, food wanting, honeybee, lncRNAs

## Abstract

Food wanting in honeybees is closely associated with the neurotransmitter dopamine; however, the regulatory role of non-coding RNAs in this process remains unclear. In this study, using the honeybee (*Apis mellifera*) as a model organism, we systematically investigated the molecular network and functional mechanisms by which long non-coding RNAs (lncRNAs) regulate the dopaminergic signaling pathway to mediate food wanting. By establishing two appetite-state models, fed honeybees (FB) and starved honeybees (SB), and combining brain dopamine quantification with behavioral assays, we found that dopamine levels in the honeybee brain were significantly elevated during starvation. Using transcriptome sequencing, we identified 1146 lncRNAs in the honeybee brain, among which 174 were differentially expressed long noncoding RNAs (DElncRNAs) between the two states, predominantly upregulated. Cis- and trans-acting analyses revealed that these DElncRNAs could target multiple genes involved in neural signal transmission, synaptic function, and dopaminergic pathways. KEGG enrichment analysis showed that their target genes were significantly enriched in pathways such as taste transduction, dopaminergic synapse, and neuroactive ligand–receptor interaction. Furthermore, a ceRNA network revealed that several DElncRNAs may regulate dopamine synthesis genes, including DOPA decarboxylase (*Ddc*), by competing for dopamine-associated miRNAs such as miR-375-3p, influencing food wanting in honeybees. Overall, our findings provide a foundation for uncovering the potential regulatory mechanisms of DElncRNAs in honeybee food wanting and offer new insights into the connection between neural regulation and behavioral manifestation in insects.

## Simple Summary

Food wanting is a critical behavior that ensures survival and supports colony-level function in honeybees (*Apis mellifera*). Although dopamine is known to modulate this behavior, the upstream molecular regulators remain largely unexplored. In this study, we show that starvation elevates brain dopamine levels and alters the expression of long non-coding RNAs (lncRNAs). Many of these lncRNAs act through cis- and trans-regulatory mechanisms and as competing endogenous RNAs (ceRNAs) to influence genes involved in dopamine synthesis, synaptic function, and neural signaling. Our findings reveal that lncRNAs modulate dopaminergic pathways to regulate food wanting, providing a new molecular perspective on food wanting regulation in social insects.

## 1. Introduction

Honeybees (*Apis mellifera*) are a key social insect model for studying genetics, behavior, and neurobiology, with division of labor, such as the transition from nurse to forager, closely linked to dynamic changes in brain gene expression [1,2]. The motivation for foraging, or food wanting, is influenced not only by individual energy states but also by colony-level demands, representing a major facet of collective behavior in social insects and directly impacting colony nutrient allocation and ecological function [3,4]. The dopaminergic signaling pathway has been shown to regulate food wanting in honeybees, providing important insights into the neural mechanisms underlying motivational behavior in insects [5]. Long non-coding RNAs (lncRNAs), abundantly transcribed but non-protein-coding, play key roles in gene expression regulation and can influence neural development, synaptic function, and behavioral plasticity through chromatin remodeling, transcriptional control, or acting as competing endogenous RNAs (ceRNAs) that bind miRNAs [6,7,8]. However, the functions of lncRNAs in regulating honeybee food wanting and dopaminergic signaling remain largely unexplored, highlighting the importance of investigating their potential roles in modulating both individual motivation and colony-level foraging behavior.

lncRNAs are a class of RNA molecules longer than 200 nucleotides, typically multi-exonic and non-protein-coding, with highly tissue- and stage-specific expression patterns [9]. Once regarded as “genomic dark matter”, they are now recognized as key regulators in development and gene expression [8,10]. lncRNAs can modulate gene expression by interacting with transcription factors and chromatin modifiers or through epigenetic regulation pathways [11,12,13]. For instance, certain antisense lncRNAs can hybridize with sense mRNAs to modulate gene silencing, transcriptional activity, or mRNA stability [14,15,16]. In cis-regulation, lncRNAs mainly affect the expression of neighboring protein-coding genes within a typical 10 kb range by modulating transcriptional activation, repression, or mRNA stability. In contrast, trans-regulation involves interactions with distant target genes, often predicted through co-expression or correlation analyses [17].

Recent studies have shown that lncRNAs play key roles in the regulation of insect behavior. In model insects such as locusts (*Locusta migratoria*), fruit flies (*Drosophila melanogaster*), and silkworms (*Bombyx mori*), numerous lncRNAs have been identified that are closely associated with neural activity and behavioral regulation [18,19,20]. In honeybees, several lncRNAs have been characterized with important neuroregulatory and behavioral functions. For instance, *kakusei*, a neural activity-associated lncRNA, is specifically activated in small-type Kenyon cells (sKCs) of the mushroom bodies and participates in the regulation of foraging behavior [21,22]. *Ks-1* influences feeding behavior by modulating neural functions in the brain [23], while *Nb-1* is tightly associated with the division of labor, particularly the transition from nurse to forager honeybees [24]. At the molecular level, kakusei regulates nearby genes such as *SCM-1* and *wupA* in cis, and modulates the expression of *Hspa8* and *gpr89* in trans, suggesting its involvement in complex social behaviors such as the waggle dance [25]. Collectively, these studies indicate that lncRNAs modulate neural plasticity to precisely regulate food choice, learning, memory, and decision-making in honeybees, ultimately contributing to the emergence of complex foraging strategies and social behaviors [21,23,26].

Notably, behavior-associated lncRNAs often interact with neurotransmitter systems. In honeybees, dopamine synthesis and signaling are essential for regulating food wanting, linking internal energy states to foraging behavior [5,27]. Recent studies in *Apis mellifera* have identified brain-expressed lncRNAs that are responsive to nutritional states and environmental stressors, suggesting their potential involvement in modulating feeding and foraging behaviors [9,28,29]. For example, RNA- analyses revealed that several lncRNAs are differentially expressed under starvation or altered colony conditions, and their putative targets include genes in the dopaminergic and metabolic pathways [9,28]. Building on this, our previous research identified that the dopamine-related gene *CYP9Q1* is differentially expressed in the heads of honeybees under different food-wanting states, suggesting its potential involvement in dopamine synthesis and regulation of food wanting [30]. Nevertheless, it remains unclear how lncRNAs contribute to honeybee food wanting, including whether they regulate dopamine synthesis, neurotransmission, or neural circuits to modulate this behavior.

This study systematically investigates how lncRNAs mediate dopaminergic regulation of food wanting in honeybees from multiple functional and molecular perspectives, and delineates the associated molecular pathways. These findings not only advance our understanding of the neurobiological basis of food wanting in honeybees but also provide new insights into the molecular links between brain function and complex behaviors.

## 2. Materials and Methods

### 2.1. Honeybees

The honeybees (*Apis mellifera*) used in this study were obtained from the “Bee Qiang No. 1” strain maintained at Fuqing Honeybee Farm and reared at Fujian Agriculture and Forestry University (FAFU), Fuzhou, China. Brood frames containing sealed pupae were first incubated under controlled conditions, and newly emerged workers were thorax-marked and returned to their original colonies. All experimental honeybees were 23-day-old foragers [5,31]. To avoid overrepresentation of any single colony, marked honeybees were collected from five colonies, with 50 individuals sampled from each colony. Marked foragers were collected 23 days after emergence for subsequent experiments, as described by Xu et al. [30].

### 2.2. Sucrose Responsiveness Assay

**Figure 1:**
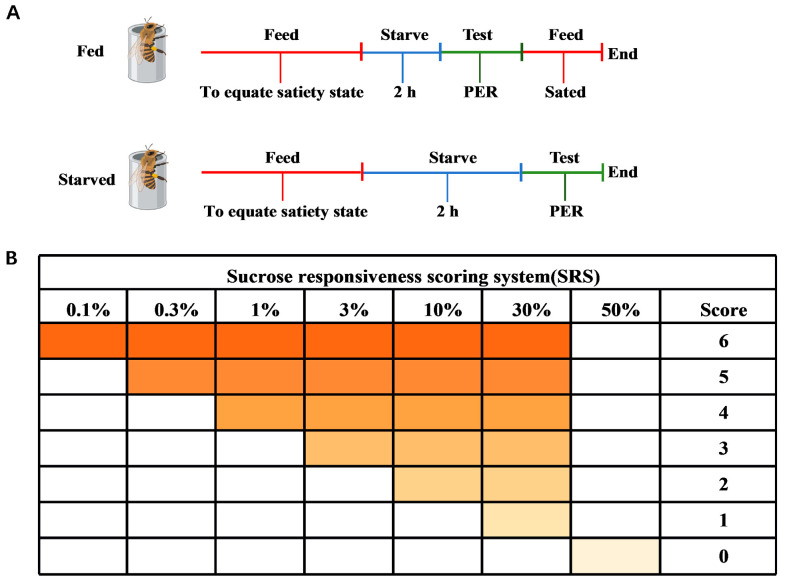
Experimental design and sucrose responsiveness in honeybees. (**A**) Schematic of sampling for fed (FB) and starved (SB) honeybees. (**B**) Sucrose responsiveness scoring system.

The sucrose responsiveness assay was performed according to the method of Huang et al. [5]. Honeybees were divided into two groups: fed honeybees (FB; honeybees starved for 2 h and then fed, serving as the control) and starved honeybees (SB; honeybees starved for 2 h) (Figure 1A). Honeybees were first anesthetized on ice for 1–2 min and then immediately transferred to copper restraining tubes for the proboscis extension response (PER) assay [30,32,33]. Food wanting was quantified using PER, a reflexive response triggered by antennal stimulation with sucrose solution. The restrained honeybees were standardized by cyclically feeding each honeybee 5 μL of ultrapure water and 50% (*w*/*w*) sucrose solution within 10 min to ensure uniform hunger and thirst levels. Before the test, honeybees were maintained in an incubator for a 2 h starvation period. Individual responsiveness was measured by presenting a series of sucrose solution concentrations (0.1, 0.3, 1, 3, 10, 30, and 50%; *w*/*w*) [34,35,36,37]. Before each sucrose solution presentation, all honeybees were tested for their response to pure water to control for the potential effects of repeated sucrose stimulations, which could have led to either sensitization or habituation. Honeybees were mounted using wooden toothpicks dipped in sugar water or ultrapure water in the following order: 0.1%, ultrapure water; 0.3%, ultrapure water; 1%, ultrapure water; 3%, ultrapure water; 10%, ultrapure water; 30%, ultrapure water; and 50%, to carry out the sucrose responsiveness assay (Figure 1B). At the end of the experiment, a sucrose responsiveness score (SRS) was obtained for each honeybee based on the sucrose concentrations to which the honeybees responded. The response was arbitrarily quantified with scores of 0 to 6, where 0 represented a honeybee that only responded to the highest sucrose concentration and 6 represented an individual that responded to all concentrations tested. Honeybees that failed to respond to any sucrose concentration were excluded from further analysis, as were honeybees that responded to all sucrose concentrations and water, since they could not discriminate between the two. Finally, qualified honeybees were rapidly frozen in liquid nitrogen and stored at −80 °C.

### 2.3. Measurement of Dopamine Levels in the Honeybee Brain

Brains (40 brains per group) were taken out of the −80 °C freezer and homogenized using a high-flux tissue homogenizer. Dopamine content in honeybee brains was quantified using a Waters Arc high-performance liquid chromatography (HPLC) system (Waters Corporation, Milford, MA, USA) coupled with a 3465 electrochemical detector (ECD), following established methods [5]. The column temperature was maintained at 40 °C, and the mobile phase flow rate was set at 0.25 mL/min. Detection was performed at 350 mV. Two measurements were taken from each sample, and 40 samples were tested for each treatment. Dopamine was quantified from single-brain tissue samples.

Neurotransmitter concentrations were quantified using an external standard method. Dopamine standards (Merck, Darmstadt, Germany) were prepared at 1 µg/mL in ultrapure water and serially diluted to 100, 80, 60, 40, 20, 10, and 5 ng/mL. Dopamine concentrations were calculated based on the peak areas of the standards using the generated standard curve and expressed as ng per brain.

### 2.4. RNA-Seq and lncRNA Identification

Total RNA was extracted from the brains of FB and SB groups (20 brains per sample, three biological replicates per group). For each group, 20 brains were pooled to form one sample, and three biological replicates were prepared. A total of 120 23-day-old worker honeybees were used for RNA sequencing. RNA extraction, cDNA library construction, and Illumina sequencing were performed by Guangzhou Gene Denovo Biotechnology Co., Ltd. (Guangzhou, China). The RNA sequencing data from this study have been deposited in the NCBI Sequence Read Archive (SRA) (http://www.ncbi.nlm.nih.gov/sra/, accessed on 18 November 2025) under BioProject accession number PRJNA1310410.

To assess overall transcriptional differences between FB and SB groups and evaluate sample repeatability, principal component analysis (PCA) was performed using the Omicsmart online platform (https://www.omicsmart.com/, accessed on 23 November 2025) with default parameters. PCA was based on normalized TPM expression matrices, and the first two principal components (PC1 and PC2) were used to visualize sample clustering. For lncRNA prediction and characterization, transcript assemblies were generated using StringTie, retaining transcripts ≥ 200 bp with at least two exons. Coding potential was evaluated using CPC2 (version 0.9), CNCI (version 2.0), and FEELnc (version 0.2) with default parameter, and the intersection of transcripts predicted as noncoding by all three tools was defined as the high-confidence lncRNA set [38]. Expression levels of all lncRNAs were normalized using the TPM (Transcripts Per Million) method. The Omicsmart online analysis platform (https://www.omicsmart.com/, accessed on 1 October 2025) was used to perform Venn analyses to identify specific and shared lncRNAs between the two groups. For structural characterization, predicted lncRNAs and mRNAs were compared through a comprehensive comparative genomic analysis, including statistical evaluation of transcript length, intron length and number, and exon length and number. All statistical analyses and visualizations were performed using GraphPad Prism (version 10.2.3) (San Diego, CA, USA).

### 2.5. Analysis of Differentially Expressed lncRNAs (DElncRNAs)

Differential expression analysis of lncRNAs between FB and SB samples was performed using the DESeq2 (version 1.20.0) package [39]. Differentially expressed lncRNAs (DElncRNAs) were identified based on the criteria of |log_2_FC| > 1.5 and *p* < 0.05. Expression clustering of the identified DElncRNAs was subsequently performed using the Omicsmart online platform (https://www.omicsmart.com/, accessed on 1 October 2025) with default parameters. Additional information on the software is available from the Bioconductor database (https://www.bioconductor.org/packages/release/bioc/, accessed on accessed on 1 October 2025).

### 2.6. Validation of DElncRNAs by RT-qPCR

To validate the reliability of the RNA-seq data, ten DElncRNAs (including MSTRG.3703.1 and XR_003305349.1) were randomly selected for RT-qPCR verification. Total RNA was extracted using the TRIzol reagent (Invitrogen, Carlsbad, CA, USA), and genomic DNA was removed and cDNA was synthesized using the HiScript^®^ II Q RT SuperMix (Vazyme, Nanjing, China). Gene-specific primers were designed with Primer Premier 6.0 and synthesized by Sangon Biotech Co., Ltd. (Shanghai, China). (Table S1). The actin gene was used as an internal reference [40]. qPCR was performed using the ChamQ Universal SYBR qPCR Master Mix (Vazyme, Nanjing, China) on a Bio-Rad C1000 Touch™ Thermal Cycler (Hercules, CA, USA). Each 20 μL reaction contained 10 μL of 2× ChamQ SYBR qPCR Master Mix, 0.4 μL of each primer (10 μM), 1 μL of cDNA template, and 8.2 μL of ddH_2_O. The cycling conditions were as follows: 95 °C for 1 min, followed by 40 cycles of 95 °C for 15 s and 60 °C for 30 s, with a final melting curve analysis from 65 °C to 95 °C at a ramp rate of 0.5 °C per 5 s. Each sample was run in triplicate, and relative expression levels of DElncRNAs were calculated using the 2^−ΔΔCT^ method [41]. All experimental procedures strictly adhered to the MIQE guidelines [42].

### 2.7. Analysis of Cis- and Trans-Acting Effects of DElncRNAs

Based on the method of Feng et al. [25], genes located within 10 kb upstream or downstream of each DElncRNA were identified as potential cis-acting targets. This 10 kb window was selected based on the average gene density and intergenic distances in the honeybee genome (*Apis mellifera*, Amel_HAv3.1), which is consistent with previous studies in insects [25,28,40]. Candidate genes were aligned using the NCBI BLAST (version 2.17.0) tool and subsequently annotated for Gene Ontology (GO) functions and Kyoto Encyclopedia of Genes and Genomes (KEGG) pathways.

For trans-acting effects, the Pearson correlation coefficient method was used to analyze correlations between lncRNAs and protein-coding genes across samples. For each lncRNA, only the gene pairs with the strongest positive and negative correlations were retained as potential trans-acting targets, ensuring a stringent selection of biologically relevant interactions. In addition, protein–protein interaction (PPI) networks of differentially expressed genes were analyzed using the STRING database (version 11.5) (http://www.string-db.org, accessed on 1 October 2025) [43], and network visualization was performed with Cytoscape (version 3.10.3) [44]. Coexpressed mRNAs were also subjected to GO and KEGG enrichment analyses.

### 2.8. Construction of the ceRNA Network

RNAplex (version 0.3) software was used to predict potential targeting relationships between DElncRNAs and DEmiRNAs, as well as between DEmiRNAs and DEmRNAs. Based on these predictions, DElncRNA-DEmiRNA-DEmRNA regulatory networks were constructed and visualized using Cytoscape (version 3.10.3) [44].

### 2.9. Statistical Analysis

Statistical analyses were performed using GraphPad Prism (version 10.2.3) (GraphPad Software Inc., San Diego, CA, USA). Data are presented as mean ± SEM, with specific details provided in the corresponding figure legends. Comparisons between two groups were conducted using Student’s two-tailed *t*-test. Statistical significance was indicated as follows: n.s., not significant; * *p* < 0.05, ** *p* < 0.01, *** *p* < 0.001, and **** *p* < 0.0001.

## 3. Results

### 3.1. Starvation Elevates Dopamine and Modulates Food Wanting in Honeybees

**Figure 2:**
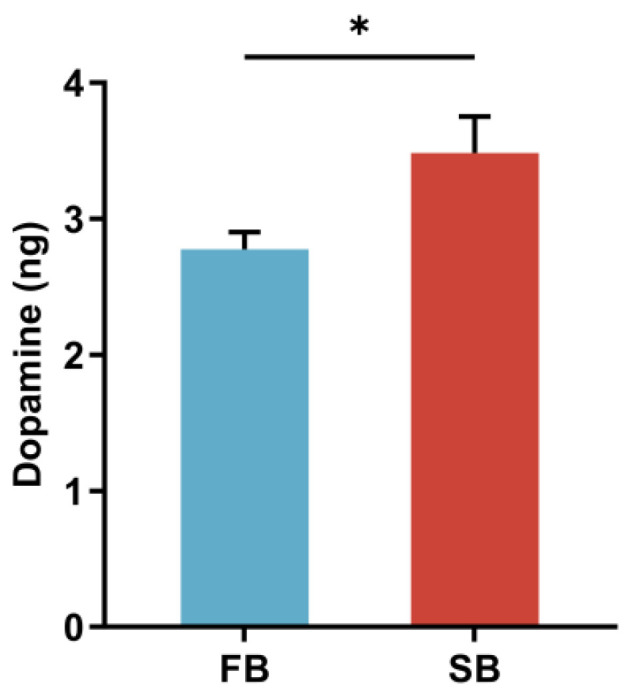
Dopamine levels in the brains of fed honeybees (FB) and starved honeybees (SB) measured by HPLC-ECD (*n* ≥ 35). Data are presented as means ± SEM. The error bars represent SEMs. * *p* ≤ 0.05.

Based on our previous findings that dopamine regulates food wanting in honeybees, we further investigated the relationship between dopamine and food wanting under starvation [5]. To this end, we established two motivational states: a fed group (FB) and a starved group (SB), and selected qualified samples based on SRS. We found that brain dopamine levels increased when honeybees transitioned from the fed to the starved state (Figure 2).

### 3.2. Sequencing and Quality Control

High-throughput sequencing generated a total of 84.3 billion raw reads (bp) across six libraries, with SB-3 yielding the highest and FB-2 the lowest read counts (Table 1). After filtering low-quality reads, 98.24–98.90% of the data were retained, meeting the required standards. The quality-controlled data were of high quality, with Q20 and Q30 scores reaching 98.48% and 95.30%, respectively (Table 1). These results indicate that the filtered data are reliable and suitable for subsequent bioinformatic analyses.

### 3.3. Number and Structural Features of lncRNAs

**Figure 3:**
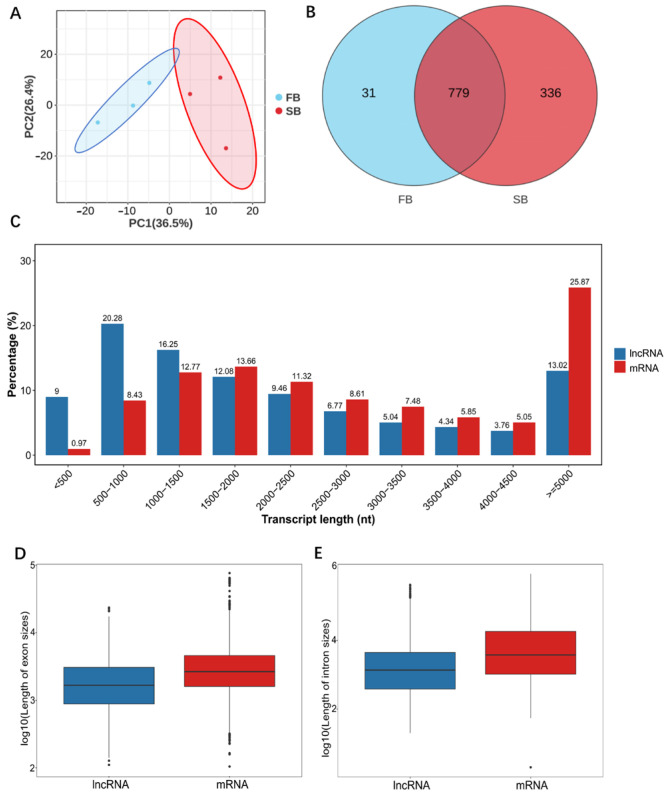
Structural features of lncRNAs and mRNAs in the FB and SB groups. (**A**) Principal component analysis (PCA) of lncRNA expression profiles in FB and SB groups. (**B**) Venn diagram of lncRNAs in the two groups. (**C**) Total intron length of lncRNAs and mRNAs. (**D**) Total exon length of lncRNAs and mRNAs. (**E**) Length distribution of lncRNAs and mRNAs.

Based on the lncRNA-seq results, principal component analysis (PCA) revealed clear segregation between the fed (FB) and starved (SB) groups, indicating distinct lncRNA expression profiles under different food-wanting states (Figure 3A). A total of 1146 lncRNAs were detected in the brains of honeybees from the FB and SB groups, with 810 and 1115 lncRNAs identified in each group, respectively. Among these, 31 and 336 lncRNAs were unique to each group (Figure 3B). Known lncRNAs accounted for 205 and 332 in the two groups, while 605 and 783 were newly predicted. The three most highly expressed lncRNAs were MSTRG.5166.10, MSTRG.6100.1, and MSTRG.8900.2. After removing redundant transcripts, a final set of 4123 lncRNAs was obtained, with lengths ranging from 192 to 416,592 nt (Figure 3C). Further structural analyses revealed that both intron and exon lengths of lncRNAs were significantly shorter than those of mRNAs (Figure 3D,E). Most lncRNAs contained 1–4 introns, accounting for approximately 80.62% (Figure S2A), while lncRNAs with 1–4 exons comprised roughly 71.84% of the total (Figure S2B).

### 3.4. RT-qPCR Validation of DElncRNAs

**Figure 4:**
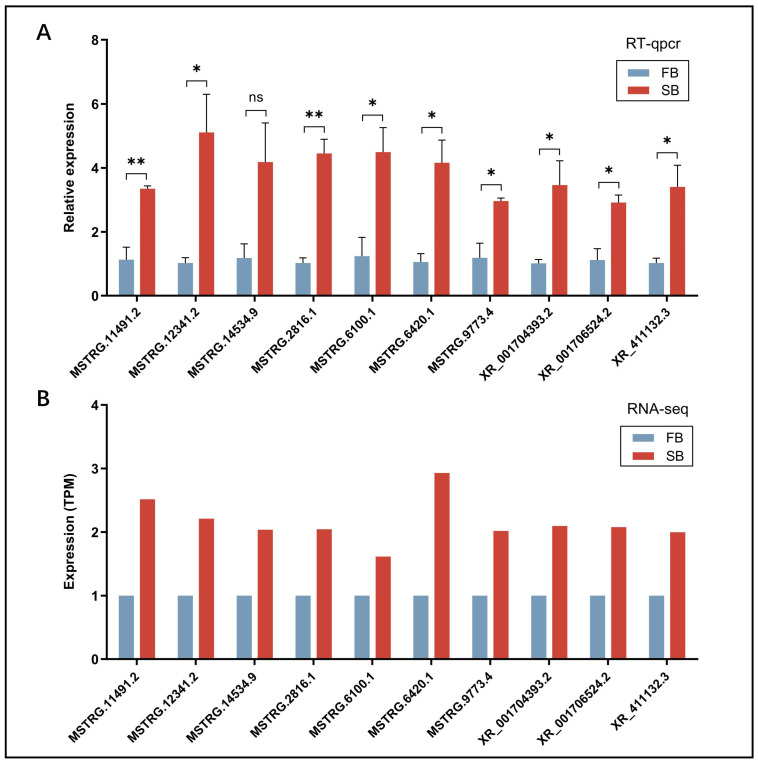
(**A**) RT-qPCR validation of the expression levels of ten randomly selected DElncRNAs. (**B**) RNA-seq expression profiles of the same ten DElncRNAs. ns, not significant; * *p* < 0.05, ** *p* < 0.01.

To verify primer specificity, all primers used for RT-qPCR were tested via amplification (of target DElncRNA fragments) and analyzed by agarose gel electrophoresis. Each primer pair produced a single band of the expected size, confirming amplification specificity (Figure S3). Subsequently, 10 randomly selected DElncRNAs were subjected to RT-qPCR validation. Quantitative analysis showed that the changes in expression of these DElncRNAs were highly consistent with the RNA-seq results (Figure 4). This validation experiment collectively ensured the reliability and accuracy of the transcriptomic data obtained in this study.

### 3.5. Differential Expression Profiles of Brain lncRNAs in Honeybee Workers Under Different Food Wanting

**Figure 5:**
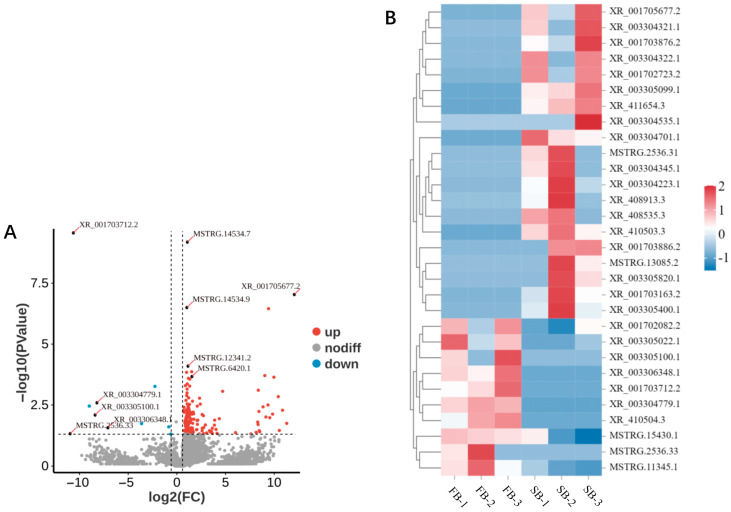
Differential expression profiles of lncRNAs in the brains of FB and SB honeybees. (**A**) Volcano plot of DElncRNAs between FB and SB groups. (**B**) Expression clustering of upregulated and downregulated lncRNAs in FB and SB groups.

Differential expression analysis of lncRNAs was performed using DESeq2, with thresholds set at *p* < 0.05 and |log2FC| > 1.5 to identify DElncRNAs. In the brains of FB and SB, a total of 174 DElncRNAs were identified, with 164 upregulated and 10 downregulated (Figure 5A,B). The five most significantly upregulated lncRNAs were XR_001705677.2 (log2FC = 12.07), MSTRG.2536.31 (log2FC = 11.29), MSTRG.13085.2 (log2FC = 10.86), XR_003304535.1 (log2FC = 10.53), and XR_003304322. 1 (log2FC = 10.42). In contrast, the five most significantly downregulated lncRNAs were MSTRG.2536.33 (log2FC = −10.97), XR_001703712.2 (log2FC = −10.63), XR_410504.3 (log2FC = −9.00), XR_003305100.1 (log2FC = −8.40), and XR_003304779.1 (log2FC = −8.21). These results indicate that transitioning from a fed to a starved state induces substantial changes in the brain lncRNA expression profile, predominantly in the form of upregulation. Given the known molecular functions of lncRNAs, such as acting as competing endogenous RNAs (ceRNAs) to bind miRNAs and thereby relieve inhibition of their target genes, these significantly upregulated lncRNAs may play crucial roles in the regulation of food wanting in honeybees. A full list of DElncRNAs is provided in Table S2.

### 3.6. GO and KEGG Analysis of Target Genes Regulated by DElncRNAs in the Brains of Honeybee Workers Under Different Food Wanting

**Figure 6:**
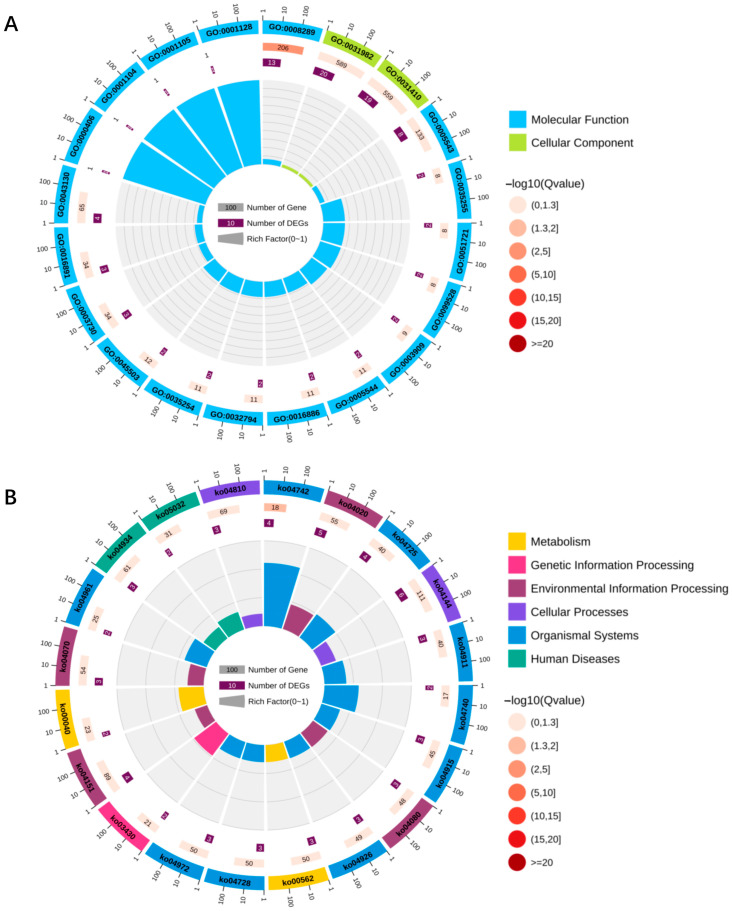
Upstream and downstream genes of the top 20 cis-regulatory DElncRNAs in fed honeybees (FB) and starved honeybees (SB) groups. (**A**) GO classification; (**B**) KEGG classification.

Accumulating evidence indicates that lncRNAs can regulate the expression of neighboring genes through cis-acting mechanisms [45]. GO annotation revealed that, via cis-regulation, 88 DElncRNAs in FB and SB potentially modulate 138 upstream and downstream genes, which are mainly involved in Cellular Components and Biological Processes (Figure 6A, Table S3). To explore the potential functions of lncRNAs in the transition between fed and starved states in worker honeybees, KEGG pathway enrichment analysis was performed on the cis-regulated target genes. The results showed that these upstream and downstream genes were significantly enriched in 146 pathways, including gustatory transduction, dopaminergic synapse, and neuroactive ligand–receptor interaction—pathways closely associated with neural regulation and food wanting (Figure 6B, Table S4). These findings suggest that lncRNAs may play important roles in modulating food wanting in worker honeybees by participating in key neural signaling pathways.

Many lncRNAs also function through trans-regulatory mechanisms, relocating to distal genomic regions and interacting with other molecules to regulate unlinked target genes [45]. Comparative analysis revealed that among the 174 DElncRNAs identified in the FB-vs-SB comparison, 157 may target 4000 genes via trans-regulatory mechanisms. GO enrichment analysis showed that these genes are primarily associated with synapse-related cellular components and are significantly enriched in biological processes such as signal transduction, regulation of biological processes, and nervous system development (Figure S4A). Furthermore, the top 20 KEGG pathways in both groups were largely related to signal transduction and nervous system functions (Figure S4B).

### 3.7. ceRNA Regulatory Network Analysis of DElncRNAs in Honeybee Brains Under Different Feeding States

**Figure 7:**
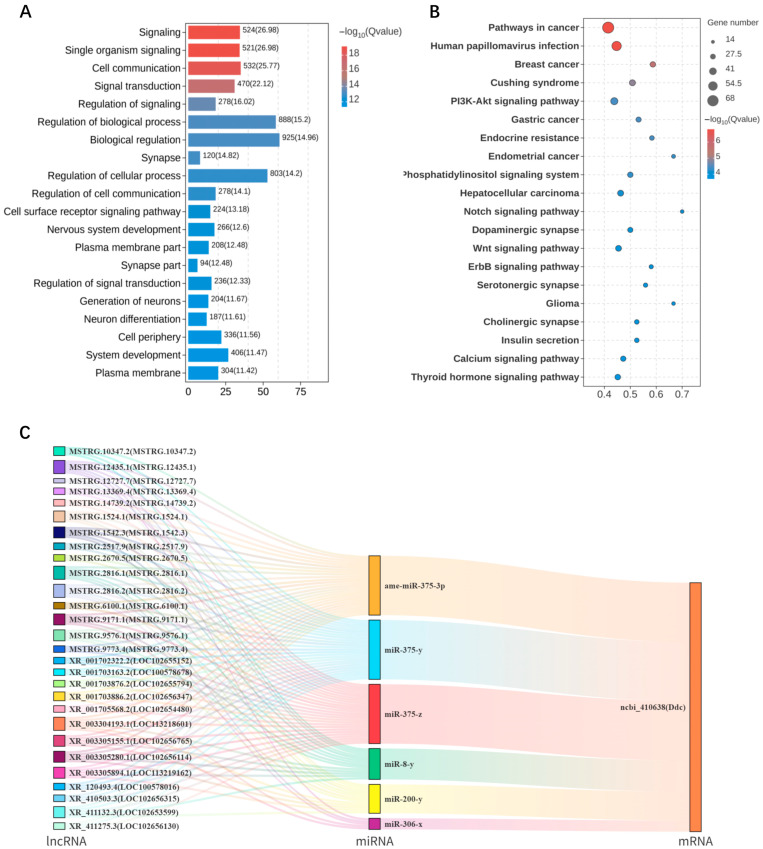
ceRNA regulatory network analysis of DElncRNAs between FB and SB groups. (**A**) Top 20 significantly enriched GO terms. (**B**) Top 20 significantly enriched KEGG pathways. (**C**) ceRNA regulatory network centered on *Ddc*.

Multiple RNA species, including lncRNAs, can modulate downstream gene expression by competitively binding miRNAs, thereby influencing biological processes such as host behavior, which is a mechanism known as competing endogenous RNA (ceRNA) regulation [19]. GO enrichment analysis revealed that the target genes involved in this network are mainly associated with synapse, plasma membrane, and cell periphery in the cellular component category, and are significantly enriched in biological processes such as signal transduction, regulation of biological processes, and nervous system development (Figure 7A). KEGG pathway analysis revealed that the top 20 enriched pathways were mainly associated with neural and hormonal signaling, including dopaminergic, cholinergic, and calcium signaling pathway, as well as PI3K-Akt, Notch, and Wnt pathways (Figure 7B). Integrating RNA-seq data with RT-qPCR validation, we identified ncbi_410638 (*Ddc*), a key gene involved in dopamine synthesis, as a potential regulator of food wanting. Based on this finding, a ceRNA regulatory network centered on *Ddc* was constructed (Figure 7C). The analysis showed that 28 lncRNAs, including MSTRG.10347.2, may simultaneously target six miRNAs such as ame-miR-375-3p, which in turn co-regulate the expression of *Ddc*. The detailed targeting relationships among DElncRNAs, DEmiRNAs, and DEmRNAs in FB and SB groups are provided in Table S5.

## 4. Discussion

Feeding motivation serves as a key neural regulatory process linking internal energy states with external foraging behavior. Dopamine has been identified as the core neurotransmitter regulating food wanting in honeybees; however, the upstream molecular mechanisms, particularly the involvement of lncRNAs, remain poorly understood [5]. Our previous research demonstrated that dopamine levels increase under starvation, accompanied by enhanced food wanting in honeybees [5,30]. Building on this, we performed transcriptome sequencing of honeybee brains under distinct food-wanting states [5]. By integrating behavioral assays, brain transcriptomics, and ceRNA network analysis, we systematically investigated the potential roles of lncRNAs in regulating food wanting. We identified numerous lncRNAs that were differentially expressed between fed and starved states, most of which were closely associated with neural signaling and synaptic function. Notably, specific lncRNAs may competitively bind dopamine-associated miRNAs such as ame-miR-375-3p to regulate dopamine synthesis, thereby modulating dopaminergic signaling and food wanting. Together, these findings suggest that lncRNAs may participate in the regulation of dopamine synthesis and signaling, providing new insights into the molecular mechanisms underlying food wanting in honeybees.

This study systematically identified lncRNAs expressed in the honeybee brain and found that their expression profiles changed markedly across different food-wanting states, with the majority of DElncRNAs being upregulated. Both cis- and trans-regulatory analyses indicated that the potential target genes of these DElncRNAs were primarily enriched in structures such as synapses, plasma membranes, and the cell periphery, as well as in Biological Processes related to signal transduction and neural development. Although many studies in mammals and model insects have demonstrated that lncRNAs influence neural activity and behavior [46,47,48,49,50], our findings extend these observations to a social insect and emphasize that starvation-induced lncRNA activation may serve as a molecular mechanism linking nutritional state to food wanting in honeybees.

Interestingly, most DElncRNAs identified here were significantly upregulated under starvation. This pattern may reflect an increased demand for signal transduction within neural circuits as energy states change. Previous studies have shown that lncRNAs can modulate synaptic plasticity, neurotransmitter release, and receptor expression, thereby enabling rapid behavioral adaptation to internal physiological states [51]. In social insects like honeybees, food wanting is tightly linked to division of labor and colony homeostasis. Thus, lncRNAs may function as molecular intermediaries linking hunger signals to neural regulation, allowing individuals to rapidly adjust behavior in response to nutritional cues and maintain colony-level food balance.

Further construction of a ceRNA regulatory network revealed that 28 DElncRNAs, including MSTRG.10347.2, may competitively bind miRNAs such as ame-miR-375-3p, thereby indirectly regulating the dopamine synthesis gene *Ddc* and modulating dopaminergic signaling. Previous studies have demonstrated that miRNAs are deeply involved in energy metabolism, neural development, and behavioral regulation in both mammals and insects [52,53]. In honeybees, we observed that specific lncRNA-miRNA-mRNA interactions, including MSTRG.10347.2 binding to ame-miR-375-3p to regulate *Ddc*, corresponded with changes in dopaminergic signaling and food wanting. These findings suggest that lncRNA-miRNA-mRNA interactions may play a critical role in regulating dopaminergic signaling and food wanting in honeybees. KEGG pathway analysis revealed significant enrichment of dopaminergic, cholinergic, and calcium signaling pathways, all of which have been implicated in insect reward and feeding circuits [54,55,56]. Moreover, DElncRNA target genes were also enriched in the PI3K-Akt, Notch, and Wnt pathways, which are canonical regulators of neural development, metabolism, and oncogenesis in mammals [57,58], and of learning, memory, and metabolic modulation in insects [20]. These findings suggest that food wanting in honeybees is not solely governed by the dopaminergic pathway, but is likely modulated through the coordinated activity of multiple signaling pathways.

In conclusion, our findings provide the first evidence that lncRNAs can modulate dopaminergic signaling via a ceRNA mechanism to regulate food wanting in honeybees, offering a new molecular perspective on how internal energy states shape foraging-related behaviors in social insects. Notably, the starvation-induced activation of lncRNAs observed here parallels patterns reported in other insects and vertebrates, where lncRNA-ceRNA-mRNA networks regulate dopaminergic function, metabolic homeostasis, and motivational states [20,59]. Such cross-species similarities suggest that lncRNA-mediated modulation of feeding motivation represents an evolutionarily conserved regulatory strategy [60,61]. As highly social insects, honeybees’ food wanting not only affects individual foraging flexibility but also shapes colony-level nutrient allocation and task efficiency. Understanding these lncRNA-based regulatory mechanisms may ultimately provide new molecular indicators for assessing colony nutritional stress, thereby contributing to honeybee health and long-term population resilience.

Nevertheless, this study has several limitations. First, although our data provide strong correlative evidence, the functional roles of specific lncRNAs in regulating food wanting remain to be experimentally validated. In future studies, we plan to focus on key DElncRNAs and perform targeted knockdown experiments using RNAi or CRISPR-based approaches, followed by behavioral assays and molecular analyses to assess their effects on dopaminergic signaling and food wanting. Second, while we identified the potential involvement of signaling pathways such as PI3K-Akt, Notch, and Wnt in behavioral regulation, the interactions among these pathways have not been fully explored. Future studies should build on these findings to further elucidate the functional roles and mechanisms of lncRNAs in the regulation of complex behaviors in social insects.

## 5. Conclusions

This study successfully uncovered the regulatory mechanisms of food wanting in honeybees by establishing distinct physiological states. For the first time, we profiled lncRNA expression in the honeybee brain under different food-wanting states using RNA-seq and examined the molecular basis of food wanting through lncRNA-ceRNA-mRNA regulatory networks. We found that the dopaminergic signaling pathway plays a central role in appetite regulation, with brain dopamine levels markedly elevated under starvation and accompanied by widespread changes in lncRNA expression. These lncRNAs participate in neural signaling and synaptic function through cis- and trans-regulatory actions as well as ceRNA mechanisms, highlighting the tight coupling between behavior, gene expression, and neural activity. This study thus offers a new theoretical framework and conceptual approach for investigating the neuro-molecular mechanisms of food wanting.

## Figures and Tables

**Table 1:** Summary of data quality control.

Sample	RawData (bp)	CleanData (bp)	Q20 (%)	Q30 (%)	GC (%)
FB-1	12,505,123,800	12,284,662,685	98.72%	95.82%	41.92%
FB-2	13,379,102,100	13,181,083,364	98.46%	95.34%	42.50%
FB-3	14,617,708,800	14,365,114,816	98.43%	95.25%	41.76%
SB-1	12,632,474,400	12,453,174,406	98.29%	94.81%	42.06%
SB-2	14,448,514,500	14,242,726,672	98.30%	94.85%	41.12%
SB-3	16,758,015,300	16,573,890,889	98.69%	95.71%	40.83%

Note: FB represents fed honeybees, SB represents starved honeybees.

## Data Availability

The data that support the findings of this study are available from the corresponding author upon reasonable request.

## Supplementary Materials

The following supporting information can be downloaded at: https://www.mdpi.com/article/10.3390/insects16121214/s1, Figure S1: Schematic diagram of the sucrose responsiveness assay. Figure S2: (A) Statistics of intron numbers in lncRNAs and mRNAs; (B) Statistics of exon numbers in lncRNAs and mRNAs. Figure S3: Agarose gel electrophoresis of RT-qPCR products amplified from lncRNAs. M: DNA marker; Lanes 1–10: Experimentally validated lncRNAs by RT-qPCR. Figure S4: (A) Top 20 GO terms annotated by the upstream and downstream genes of DElncRNAs in FB vs. SB. (B) Top 20 KEGG pathways annotated by the upstream and downstream genes of DElncRNAs in FB vs. SB. Table S1: Primers used in the qPCR analysis. Table S2: The complete list of differentially expressed lncRNAs (DElncRNAs) in the brains of FB (fed honeybees) and SB (starved honeybees). Table S3: Top 20 classes annotated by upstream and downstream genes of DElncRNAs in FB vs. SB. Table S4: Top 20 pathways annotated by upstream and downstream genes of DElncRNAs in FB vs. SB. Table S5: Detailed interactions within the ceRNA network: DElncRNAs, DEmiRNAs, and their target gene *Ddc*.
