# Supplementary material for: Role of Long Non-Coding RNAs in Food Wanting of Apis Mellifera
Source: Insects. 2025 Nov 28;16(12):1214. doi: 10.3390/insects16121214 (PMC12734153; doi:10.3390/insects16121214)
Supplement: Supplementary file 1 [file insects-16-01214-s001.zip › Supplementary Materials/Figure S3.pdf]

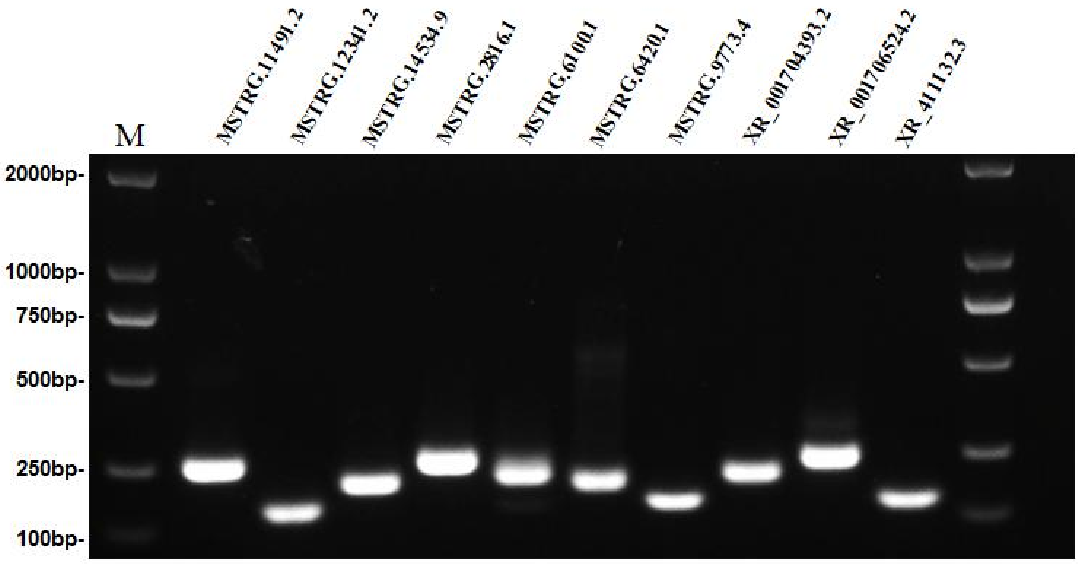

Figure S3. Agarose gel electrophoresis of RT-qPCR products amplified from lncRNA . M: DNA marker; Lane 1-10: Experimentally validated lncRNAs by RT-qPCR.
