# Supplementary material for: Role of Long Non-Coding RNAs in Food Wanting of Apis Mellifera
Source: Insects. 2025 Nov 28;16(12):1214. doi: 10.3390/insects16121214 (PMC12734153; doi:10.3390/insects16121214)
Supplement: Supplementary file 1 [file insects-16-01214-s001.zip › Supplementary Materials/Table S1.pdf]

Table S1. Primers used in the qPCR analysis

LncRNA Name	Primer Sequences (5′→3′)	Product Size (bp)
MSTRG.11491.2-F	GTGACGATGGTACGACAATTAGAC	236bp
MSTRG.11491.2-R	GAGTGGTCCGATGAGTTATGC	
MSTRG.12341.2-F	ACACAGTAGCAGCACAGATAGG	124bp
MSTRG.12341.2-R	GCAACAACCATCACGCATAGT	
MSTRG.14534.9-F	TACTTTCCCAGGTTGCGATTG	177bp
MSTRG.14534.9-R	CGATTAGCGTGACCGTTCTC	
MSTRG.2816.1-F	CGTTCCTCGGTCATCTATTCC	237bp
MSTRG.2816.1-R	TGTATTCGTCATCATCGCAAGATT	
MSTRG.2816.2-F	GCTTTGTCGATCAATACTCGGTTT	204bp
MSTRG.2816.2-R	CGTCGCAGTGCTGAAAGTTC	
MSTRG.6100.1-F	AAGACCATAGGCGATGATTAGCA	181bp
MSTRG.6100.1-R	CCAAGTCACACCACGAGGAG	
MSTRG.6420.1-F	ACCTCCTGTCGTATCATGTTCTC	130bp
MSTRG.6420.1-R	AGAGCGTCAGAATATAGCCCTTT	
MSTRG.9773.4-F	ACCTATCTCCAAAGCAAGTTTCC	202bp
MSTRG.9773.4-R	CACGGTGGCATCTCTTCATT	
XR_001704393.2-F	TTGCCTCTTGTCACACGAATG	232bp
XR_001704393.2-R	GCTCTCCGACTTCCTTTCCT	
XR_001706524.2-F	CAGACGAATGGATTGCGAAGT	110bp
XR_001706524.2-R	TCCGTATGCCTTTGTAGTAGAGT	
XR_411132.3-F	CAATATGTCTCCACTCCACTGATT	236bp
XR_411132.3-R	TGCGACGATTAGAAGAATGATTCC	
β-Actin-F	CCTAGCACCATCCACCATGAA	87bp
β-Actin-R	GAAGCAAGAATTGACCCACCAA	

***F: forward primer; R: reverse primer**
