# Supplementary material for: Role of Long Non-Coding RNAs in Food Wanting of Apis Mellifera
Source: Insects. 2025 Nov 28;16(12):1214. doi: 10.3390/insects16121214 (PMC12734153; doi:10.3390/insects16121214)
Supplement: Supplementary file 1 [file insects-16-01214-s001.zip › Supplementary Materials/Table S3.pdf]

Table S3. Top 20 GO terms annotated by the upstream and downstream genes of DElncRNAs in FB vs. SB

GO_Class	GO_ID	Number	pvalue
Cellular Component	GO:0031982	20	0.000538
Cellular Component	GO:0031410	19	0.000752
Molecular Function	GO:0008289	13	0.000008
Molecular Function	GO:0005543	8	0.000674
Molecular Function	GO:0035255	2	0.005546
Molecular Function	GO:0051721	2	0.005546
Molecular Function	GO:0099528	2	0.005546
Molecular Function	GO:0003909	2	0.007063
Molecular Function	GO:0005544	2	0.010589
Molecular Function	GO:0016886	2	0.010589
Molecular Function	GO:0032794	2	0.010589
Molecular Function	GO:0035254	2	0.010589
Molecular Function	GO:0045503	2	0.012587
Molecular Function	GO:0003730	3	0.012920
Molecular Function	GO:0016891	3	0.012920
Molecular Function	GO:0043130	4	0.014544
Molecular Function	GO:0000406	1	0.014549
Molecular Function	GO:0001104	1	0.014549
Molecular Function	GO:0001105	1	0.014549
Molecular Function	GO:0001128	1	0.014549
