# Supplementary material for: Role of Long Non-Coding RNAs in Food Wanting of Apis Mellifera
Source: Insects. 2025 Nov 28;16(12):1214. doi: 10.3390/insects16121214 (PMC12734153; doi:10.3390/insects16121214)
Supplement: Supplementary file 1 [file insects-16-01214-s001.zip › Supplementary Materials/Table S4.pdf]

Table S4. Top 20 KEGG pathways annotated by the upstream and downstream genes of DElncRNAs in FB vs. SB

KEGG_Pathway	KEGG_ID	Up	Down	Pvalue
Taste transduction	ko04742	4	0	0.000122
Calcium signaling pathway	ko04020	5	0	0.001277
Cholinergic synapse	ko04725	4	0	0.002853
Endocytosis	ko04144	6	0	0.005995
Insulin secretion	ko04911	3	0	0.021860
Olfactory transduction	ko04740	2	0	0.026524
Estrogen signaling pathway	ko04915	3	0	0.029789
Neuroactive ligand-receptor interaction	ko04080	3	0	0.035185
Relaxin signaling pathway	ko04926	3	0	0.037089
Inositol phosphate metabolism	ko00562	3	0	0.039045
Dopaminergic synapse	ko04728	3	0	0.039045
Pancreatic secretion	ko04972	3	0	0.039045
Mismatch repair	ko03430	2	0	0.039417
PI3K-Akt signaling pathway	ko04151	4	0	0.044436
tose and glucuronate interconversi	ko00040	2	0	0.046591
ıosphatidylinositol signaling syste	ko04070	3	0	0.047390
Endocrine and other factor-regulated calcium reabsorption	ko04961	2	0	0.054203
Cushing syndrome	ko04934	3	0	0.063927
Morphine addiction	ko05032	2	0	0.079368
Regulation of actin cytoskeleton	ko04810	3	0	0.085658
