# Supplementary figures and images for: Role of Long Non-Coding RNAs in Food Wanting of Apis Mellifera
Source: Insects. 2025 Nov 28;16(12):1214. doi: 10.3390/insects16121214 (PMC12734153; doi:10.3390/insects16121214)

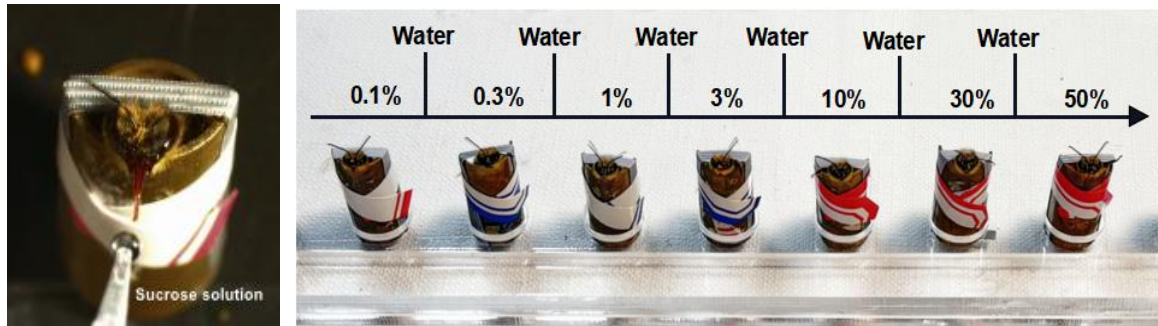

Figure S1. Schematic diagram of the sucrose responsiveness assay

Supplement: Supplementary file 1 [file insects-16-01214-s001.zip › Supplementary Materials/Figure S1.pdf]
