# Supplementary material for: Role of Long Non-Coding RNAs in Food Wanting of Apis Mellifera
Source: Insects. 2025 Nov 28;16(12):1214. doi: 10.3390/insects16121214 (PMC12734153; doi:10.3390/insects16121214)
Supplement: Supplementary file 1 [file Figure S4.pdf › Supplementary Materials/Figure S4.pdf]

A

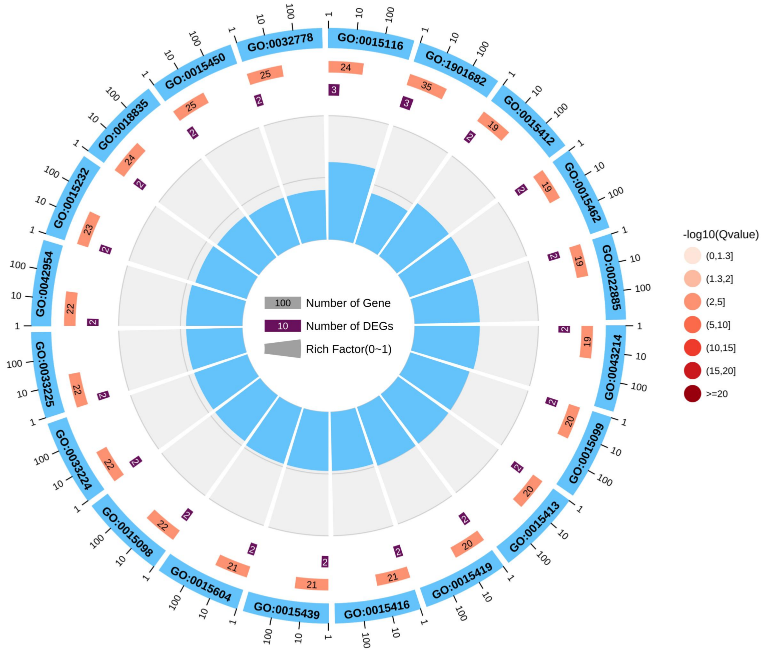

B

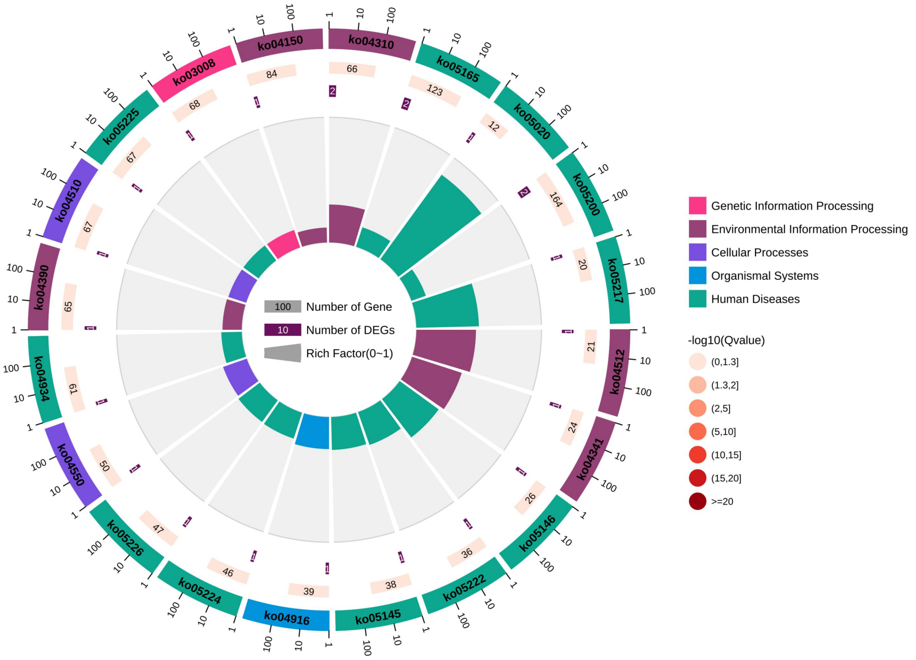

Figure S3. (A) GO classification of the upstream and downstream genes of the top 20 DElncRNAs in FB vs. SB. (B) KEGG pathway enrichment of the upstream and downstream genes of the top 20 DElncRNAs in FB vs. SB.
